# Bony Union Following Platelet-Rich Plasma and Focused Extracorporeal Shock Wave Therapy for Delayed Union of Bilateral Lumbar Spondylolysis in an Adolescent Gymnast: A Case Report

**DOI:** 10.7759/cureus.117524

**Published:** 2026-10-06

**Authors:** Leo Ishizuka, Nao Eguchi, Satoru Katsumata, Kiyoshi Yoshihara

**Affiliations:** 1 Orthopaedics, AR-Ex Spine Clinic, Tokyo, JPN; 2 Rehabilitation, AR-Ex Spine Clinic, Tokyo, JPN

**Keywords:** adolescent athlete, bony union, case report, delayed union, extracorporeal shock wave therapy, focused eswt, lumbar spondylolysis, pars interarticularis, platelet-rich plasma

## Abstract

Lumbar spondylolysis is a stress fracture of the pars interarticularis that is common in adolescent athletes. In this report, we describe the case of a 16-year-old male competitive gymnast who presented with low back pain. Magnetic resonance imaging (MRI) demonstrated bilateral L4 spondylolysis with high signal in the pars interarticularis (right, progressive stage; left, early stage). Sport was stopped completely, and a rigid thoracolumbosacral orthosis was fitted immediately. Pain improved gradually, and the high signal on fat-suppressed T2-weighted images regressed, but the fracture lines extended rather than consolidated, and computed tomography (CT) at three months showed no bony bridging on either side. The right lesion remained at the progressive stage, and the left showed uniformly sclerotic fracture margins and no bridging trabeculae, indicating that it was approaching the terminal stage. Delayed union was diagnosed. After informed consent was obtained, focused extracorporeal shock wave therapy (f-ESWT) was delivered to the bilateral pars defects in three sessions at approximately weekly intervals, and a single injection of autologous platelet-rich plasma (PRP) was administered at the defects under fluoroscopic guidance before the second session. Transient injection-site pain was the only adverse event, and no neurological complications occurred. CT at two months showed bony bridging progressing bilaterally; a graduated return to gymnastics training was begun, and CT at five months confirmed bilateral union. At nine months, the patient had trained without restriction for four to five months without recurrence of low back pain, and MRI obtained after an acute hyperextension injury showed both pars defects still united. To our knowledge, this is the first report of PRP injected at a pars interarticularis defect in lumbar spondylolysis. Because both modalities were applied concurrently in a single patient while rigid bracing was continued, their individual contributions cannot be separated, and spontaneous union cannot be excluded. Nevertheless, the course changed from stalled to healing after the intervention, in a lesion considered unfavorable for spontaneous union. This single observation is hypothesis-generating rather than evidence of treatment efficacy, and it suggests that combined PRP and f-ESWT may merit evaluation in comparative studies of delayed-union spondylolysis that has not responded to conservative treatment.

## Introduction

Lumbar spondylolysis is a stress fracture of the pars interarticularis and is one of the most common causes of low back pain in adolescent athletes engaged in repetitive lumbar extension and rotation [1,2]. The probability of bony union under conservative treatment depends strongly on the stage of the lesion at diagnosis: using a staging system based on computed tomography (CT), Ohba et al. reported that the proportion of patients showing union or a favorable course on CT at three months was 100% (11 of 11), 73.7% (14 of 19), and 40% (4 of 10) for stages I, II, and III, respectively [1]. In a separate series of children and adolescents treated conservatively with a soft brace for at least three months, no terminal-stage defect united (0 of 7) [3]. When union is not achieved, the long-term consequences differ markedly between unilateral and bilateral lesions. In a CT study of 2,000 adults in the general Japanese population, spondylolisthesis was present in 74.5% of subjects with bilateral spondylolysis but in only 7.7% of those with unilateral spondylolysis [4]. A structured literature review by the Scoliosis Research Society reached a similar conclusion, reporting that 43-74% of patients with chronic bilateral pars defects progress to grade 1 or 2 spondylolisthesis [5]. Achieving union therefore remains a clinically meaningful goal in a skeletally immature athlete with bilateral lesions.

Extracorporeal shock wave therapy (ESWT) promotes fracture healing by inducing microtrauma, growth factor release, and neovascularization [6], and has been used in the treatment of stress fractures, delayed union, and nonunion. Its application to lumbar spondylolysis has been reported in Japan, but union rates fell steeply with advancing stage, and the presence of a contralateral lesion was also associated with a lower union rate [2]. In a series of lesions that had remained ununited after three months of conservative treatment and were then treated with ESWT, only one of four bilateral cases achieved union on both sides [7]. Among those four bilateral cases, every side that failed to unite was at the terminal stage. Lesions at or approaching the terminal stage thus represent the subgroup in which ESWT alone frequently fails to achieve union.

No clinical evidence is available on combining platelet-rich plasma (PRP) with ESWT for lumbar spondylolysis. The combination has been reported to accelerate healing in aseptic nonunion of long bones [8], in nonunion of a stress fracture of the proximal phalanx of the great toe [9], and in refractory femoral nonunion [10]; none of these reports concerned lumbar spondylolysis, and their findings cannot be assumed to apply to the pars interarticularis. The rationale for the combination is that PRP supplies the growth factors whose release ESWT stimulates. In the lumbar spine, PRP has been used for radicular pain associated with spondylolisthesis [11], but we found no report of PRP injected at a pars interarticularis defect. PubMed searches performed in September 2026, combining "spondylolysis" or "pars interarticularis" with "platelet-rich plasma", "shockwave", "shock wave", or "extracorporeal therapy", returned only that single article [11]. Because ESWT for lumbar spondylolysis has been reported mainly in the Japanese-language literature, Ichushi-Web (Japan Medical Abstracts Society) was also searched in September 2026. Searches for "lumbar spondylolysis" with "platelet-rich plasma", for "pars interarticularis" with "platelet-rich plasma", and for "platelet-rich plasma" with "extracorporeal shock wave therapy" and "lumbar spine" each returned no records, and eight records retrieved for "spondylolysis" with "extracorporeal shock wave therapy" described ESWT without PRP. These searches were limited to two databases and to English- and Japanese-language publications, so reports indexed elsewhere or published in other languages may have been missed. Consistent with this, a 2022 Japanese review from a clinic that provides both PRP and ESWT as self-funded care for athletes described the combination of the two modalities as a future prospect expected to improve treatment outcomes, rather than as an established practice [12]. Here, we report a case of bilateral L4 spondylolysis with delayed union in which combined PRP injection and focused ESWT (f-ESWT) were followed by bilateral bony union and full return to unrestricted gymnastics training.

This article was previously presented orally at the Annual Meeting of the Japan Sports Orthopaedic Association on August 27, 2026.

## Case presentation

A 16-year-old male competitive gymnast with 12 years of gymnastics experience, training five days per week, presented with low back pain. The pain had begun approximately two weeks before presentation, during gymnastics practice, without a specific inciting event. There was no history of endocrine or metabolic bone disease, and he was taking no medication affecting bone metabolism.

On examination, there was tenderness over the L4 region and pain on lumbar extension. There was no radicular pain and no sensory or motor deficit. Pain intensity, lumbar range of motion, and functional limitation were not recorded quantitatively at the initial visit. Radiographs showed a mild scoliotic curve convex to the right and no spondylolisthesis. Magnetic resonance imaging (MRI) performed at the initial visit demonstrated high signal intensity in the L4 pars interarticularis bilaterally on axial fat-suppressed T2-weighted images (Figure 1A). CT-like reconstructions were generated from a three-dimensional T1-weighted gradient-echo (volumetric interpolated breath-hold examination) sequence (repetition time 7 ms, echo time 2.46 ms, flip angle 10°, two signal averages, slice thickness 0.9 mm) (Figure 1B). These reconstructions established the diagnosis of bilateral L4 spondylolysis, progressive stage on the right (Figure 1C) and early stage on the left (Figure 1D).

**Figure 1:**
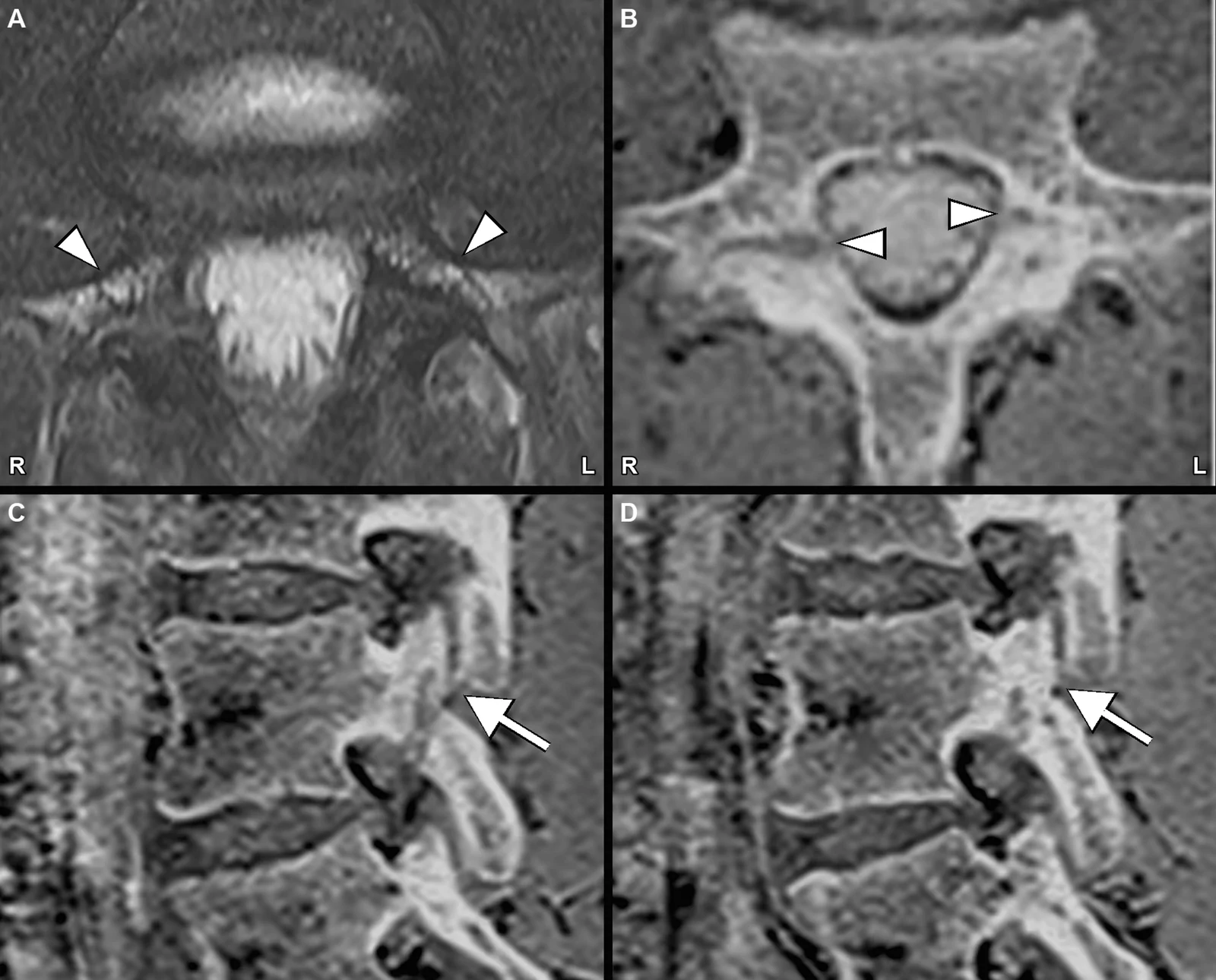
Magnetic resonance imaging at the initial visit (A) Axial fat-suppressed T2-weighted image showing high signal intensity in the L4 pars interarticularis bilaterally. (B) Axial CT-like reconstruction at the same level. (C) Right parasagittal CT-like reconstruction showing a progressive-stage lesion. (D) Left parasagittal CT-like reconstruction showing an early-stage lesion. The arrowheads in the axial images indicate the L4 pars interarticularis on each side in A and the fracture lines on each side in B, and the arrows in the parasagittal images (C, D) indicate the L4 pars interarticularis. R and L denote the patient's right and left. CT: computed tomography

Sport was stopped completely, and a rigid thoracolumbosacral orthosis was fitted immediately after the diagnosis was made. Conservative treatment was chosen first because both lesions showed high signal on fat-suppressed T2-weighted images and neither was at the terminal stage. Low back pain improved gradually with these measures. MRI was repeated at approximately two and three months after the start of conservative treatment. The high signal on the fat-suppressed T2-weighted images decreased progressively on both sides (Figure 2A-2C), but on CT-like reconstructions, the fracture lines had extended rather than consolidated on the right (Figure 2D-2F) and on the left (Figure 2G-2I).

**Figure 2:**
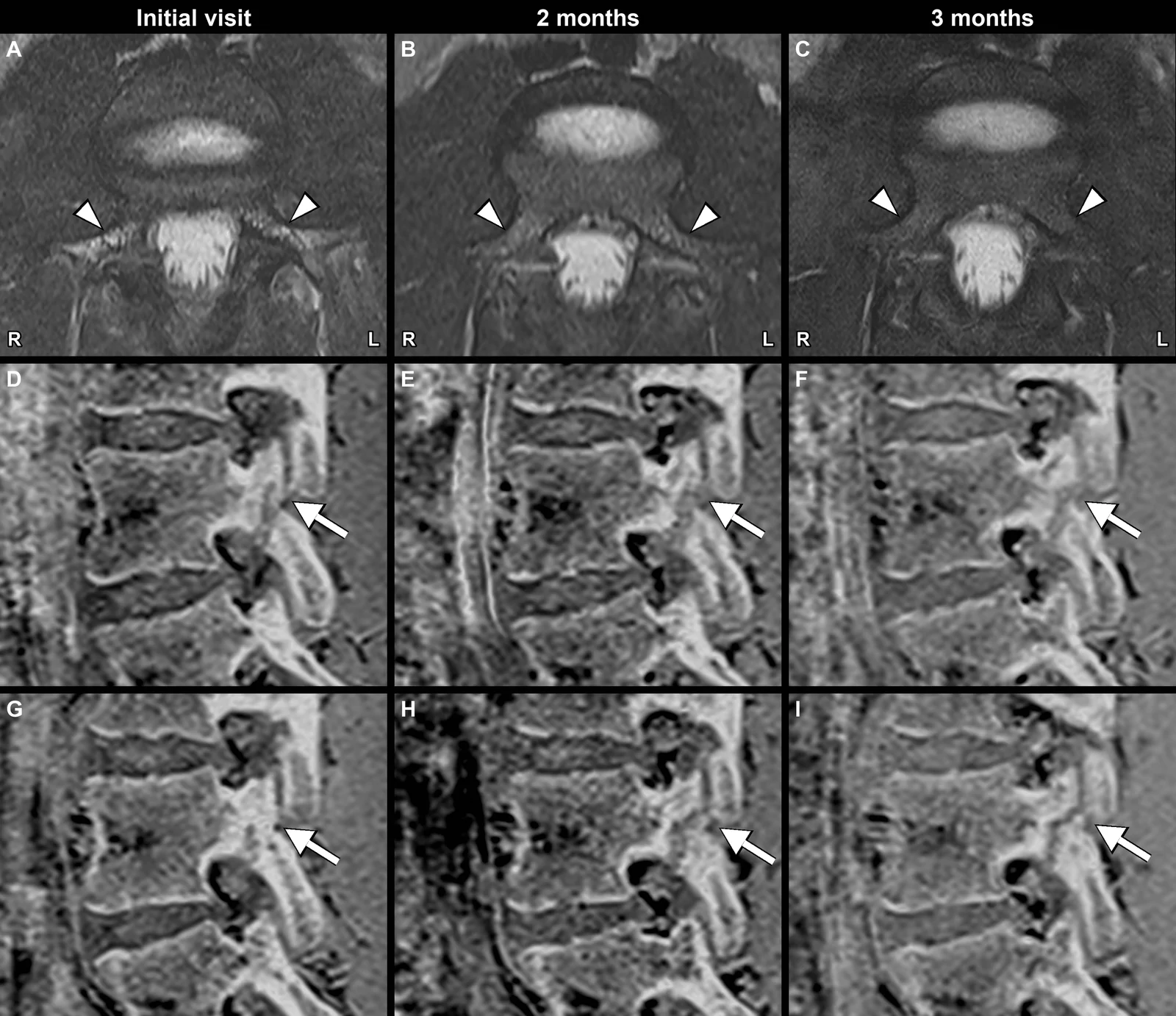
Serial magnetic resonance imaging during conservative treatment (A) Axial fat-suppressed T2-weighted image at the initial visit. (B) The same sequence at approximately two months after the start of conservative treatment. (C) The same sequence at approximately three months. (D) Right parasagittal CT-like reconstruction at the initial visit. (E) The same reconstruction at two months. (F) The same reconstruction at three months. (G) Left parasagittal CT-like reconstruction at the initial visit. (H) The same reconstruction at two months. (I) The same reconstruction at three months. All images are at the level of the L4 pars interarticularis; the arrowheads in the axial images (A-C) and the arrows in the parasagittal images (D-I) indicate the L4 pars interarticularis. R and L denote the patient's right and left. From A to C the high signal on the fat-suppressed T2-weighted images decreased progressively on both sides, whereas from D to F on the right and from G to I on the left the fracture lines extended rather than consolidated. CT: computed tomography

CT performed after approximately three months of complete cessation of sport and rigid bracing, at 120 kV with a reconstructed slice thickness of 1 mm, showed no bony bridging on either side (Figure 3A, 3D, 3G). Lesions were staged according to the conventional early, progressive, and terminal classification used for lumbar spondylolysis [3]. On both sides, the fracture line extended through the full thickness of the pars. On the right, the fracture margins were not sclerotic, and no callus was present; this lesion was classified as progressive. On the left, the fracture margins were uniformly sclerotic and no bridging trabeculae were present; this lesion was therefore considered to be approaching the terminal stage.

**Figure 3:**
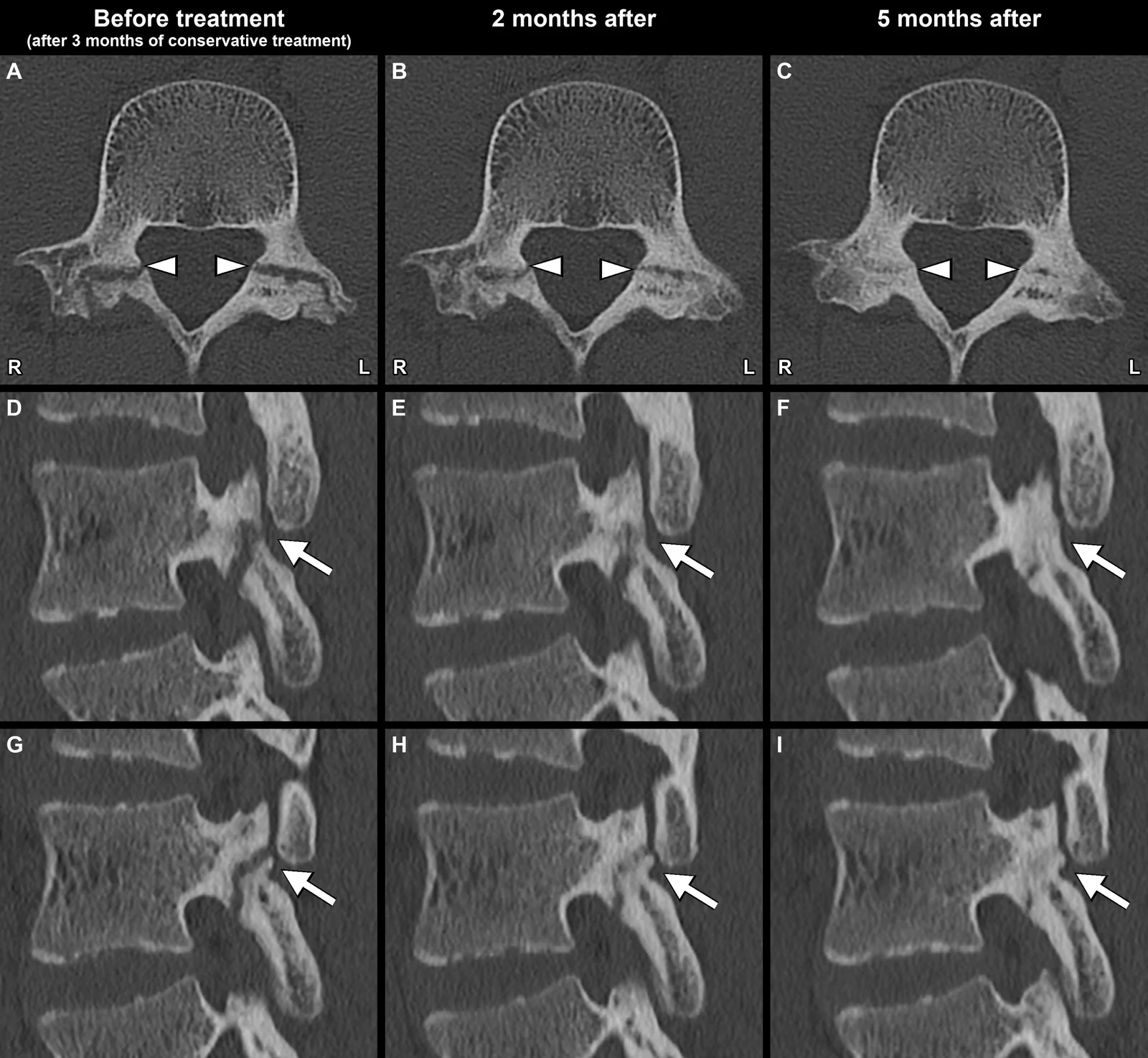
Serial computed tomography before and after combined treatment (A) Axial image before treatment, that is, after approximately three months of conservative treatment. (B) Axial image at approximately two months after the start of treatment. (C) Axial image at approximately five months after the start of treatment. (D) Right parasagittal reconstruction before treatment. (E) Right parasagittal reconstruction at two months. (F) Right parasagittal reconstruction at five months. (G) Left parasagittal reconstruction before treatment. (H) Left parasagittal reconstruction at two months. (I) Left parasagittal reconstruction at five months. All images are at the level of the L4 pars interarticularis; the arrowheads in the axial images indicate the fracture lines on each side in A and B and the sites where the fracture lines had been in C, and the arrows in the parasagittal images (D-I) indicate the L4 pars interarticularis. R and L denote the patient's right and left. No bony bridging was present on either side before treatment (D, G). Bridging had progressed bilaterally at two months (E, H). At five months, continuous trabecular bridging was present across both pars defects, with a thin residual lucency ventrally on each side (F, I). CT: computed tomography

Staging at the time of treatment was performed by the treating physician. In the absence of any radiographic progression toward union, delayed union was diagnosed. Because the left lesion, approaching the terminal stage, was unlikely to unite with continued conservative treatment [3] or with ESWT alone [7], and because bilateral pars defects that remain ununited carry a risk of progression to spondylolisthesis [4,5], additional treatment was discussed with the patient and his family. Continued conservative treatment, direct pars repair, and observation without a union goal were all presented as alternatives.

The PRP treatment was provided under a Class II regenerative medicine provision plan ("Treatment of spine-derived pain using autologous platelet-rich plasma") that was submitted to the Minister of Health, Labour and Welfare, Japan, under plan number PB3250041, in accordance with the Act on the Safety of Regenerative Medicine (Act No. 85 of November 27, 2013) [13]. The device used for f-ESWT (BTL-6000 Focus device; BTL Industries JSC, Sofia, Bulgaria) is certified in Japan only for the relief of pain in refractory plantar fasciitis that has not responded to at least six months of conservative treatment (certification number 30300BZX00272000), so its use for lumbar spondylolysis lies outside the certified intended use. Both the patient and his guardian gave written informed consent for combined PRP and f-ESWT. It was explained that the treatment was self-funded care not covered by national health insurance, that the use of f-ESWT for this condition was outside the certified intended use of the device, and that the PRP used was not an approved pharmaceutical product but was prepared and administered under the Act on the Safety of Regenerative Medicine (Japan) [13].

Treatment was begun five days after the three-month CT and was delivered in three sessions at approximately weekly intervals (Table 1). The treatment parameters are summarized in Table 2. f-ESWT was applied with a BTL-6000 Focus device at 4 Hz, 5,000 shocks per session for the two sides combined (2,500 per side). At the first session, the energy flux density was raised stepwise within the patient's tolerance, with 50 shocks delivered at 0.12 mJ/mm² and 50 at 0.22 mJ/mm² before the remainder of the session was delivered at 0.27 mJ/mm²; at the second and third sessions, all shocks were delivered at 0.27 mJ/mm². The medium coupling pad was used; this pad places the focal point at a depth of 30 mm, with a focal zone extending from 15 to 50 mm and a focus size of 5 × 5 × 35 mm.

**Table 1:** Clinical course and treatment MRI: magnetic resonance imaging; CT: computed tomography; f-ESWT: focused extracorporeal shock wave therapy; PRP: platelet-rich plasma

Time Point	Event	Findings/Details
~2 weeks before presentation	Low back pain during gymnastics practice	No specific inciting event
Initial visit	First examination	Tenderness at L4, pain on lumbar extension; no radicular signs
Initial visit	MRI	Bilateral L4 pars: high signal on fat-suppressed T2-weighted images; right, progressive stage; left, early stage
Initial visit	Conservative treatment started	Complete cessation of sport; rigid thoracolumbosacral orthosis
~2 months after initial visit	MRI	Fat-suppressed T2 high signal decreased; fracture lines extended
~3 months after initial visit	MRI	Fat-suppressed T2 high signal further decreased; no consolidation
~3 months after initial visit	CT	No bony bridging bilaterally; right, progressive stage; left, uniformly sclerotic fracture margins with no bridging trabeculae, approaching the terminal stage
Treatment week 0 (5 days after the CT)	f-ESWT session 1	5,000 shocks (both sides combined), 4 Hz; energy raised stepwise to 0.27 mJ/mm² (50 shocks at 0.12, 50 at 0.22)
Treatment week 1	PRP injection + f-ESWT session 2	PRP 6 mL at bilateral L4 pars under fluoroscopy, followed approximately 15 minutes later by f-ESWT (0.27 mJ/mm², 5,000 shocks, 4 Hz)
Treatment week 2	f-ESWT session 3	0.27 mJ/mm², 5,000 shocks (both sides combined), 4 Hz
~2 months after the start of treatment	CT	Bony bridging progressing bilaterally
~2 months after the start of treatment	Partial return to gymnastics training	Rigid orthosis continued for activities of daily living
~3 months after the start of treatment	Radiographs	Residual fissure without diastasis on both sides
~3 months after the start of treatment	Rigid orthosis discontinued	Discontinued for activities of daily living as well; training load increased. Rigid orthosis worn for approximately six months in total from the initial visit
~4 months after the start of treatment	Full return to gymnastics training begun	Lumbar extension and rotation skills reintroduced last
~5 months after the start of treatment	CT	Bilateral bony union
~5–6 months after the start of treatment	Complete routines on all six apparatus in training	No eligible competition held (off-season)
~9 months after the start of treatment	Acute low back injury during practice	Landing from the horizontal bar with forced lumbar hyperextension; no radicular or neurological signs
~9 months after the start of treatment	Radiographs and MRI	No fracture and no sign of recurrent spondylolysis; both pars defects remained united; sport not restricted

**Table 2:** Treatment parameters CT: computed tomography; f-ESWT: focused extracorporeal shock wave therapy; PRP: platelet-rich plasma

Parameter	Details
f-ESWT device	BTL-6000 Focus (BTL Industries JSC, Sofia, Bulgaria)
Energy flux density	0.27 mJ/mm² (first session: 50 shocks at 0.12 and 50 at 0.22 before the remainder at 0.27; second and third sessions entirely at 0.27)
Shocks per session	5,000 for both sides combined (2,500 per side)
Coupling pad	Medium; focal point depth 30 mm, focal zone 15–50 mm, focus size 5 × 5 × 35 mm. On CT, skin to pars approximately 45 mm on both sides, skin to posterior vertebral margin approximately 60 mm
Frequency	4 Hz
Number of sessions	3, at approximately weekly intervals
Targeting	Pars defect marked under ultrasound guidance; shock wave directed away from the spinal canal; absence of radiating leg pain confirmed
Anesthesia for f-ESWT	None
PRP system	Autologous Conditioned Plasma (ACP) Double Syringe System (Arthrex Inc., Naples, FL, USA)
Whole blood volume	15 mL
PRP volume	6 mL (leukocyte-poor); platelet concentration not measured
PRP preparation	Single centrifugation at 1,500 rpm for 5 minutes (standard protocol for the system); no anticoagulant added; not activated
PRP administration	Once, at the second session, under fluoroscopic guidance at bilateral L4 pars defects (~3 mL per side)
Sequence	PRP injection followed approximately 15 minutes later by f-ESWT at the same sites
Local anesthetic	Skin and subcutaneous needle track only; not mixed with PRP, not injected into the defect

On CT, the distance from the skin surface to the pars interarticularis measured approximately 45 mm on both sides, and the distance from the skin surface to the posterior margin of the vertebral body was approximately 60 mm. The pars defects were identified and marked under ultrasound guidance before each session. The applicator was angled from a slightly medial and caudal position, directing the shock wave laterally and cranially away from the spinal canal, and the absence of radiating leg pain was confirmed throughout each application. No anesthesia was used for f-ESWT, because intraprocedural pain served as the criterion for setting the energy level.

At the second session, PRP was administered shortly before f-ESWT; 15 mL of autologous whole blood was drawn and processed with the Autologous Conditioned Plasma (ACP) Double Syringe System (Arthrex Inc., Naples, Florida, United States) to yield 6 mL of leukocyte-poor PRP. The standard protocol for the system was followed: a single centrifugation at 1,500 rpm for five minutes, with no anticoagulant added and no exogenous activator such as calcium chloride or thrombin. Under fluoroscopic guidance, a needle was advanced to the L4 pars defect on each side, and the PRP was injected at the defect (Figure 4), approximately 3 mL per side. Needle position was confirmed in both the anteroposterior and the lateral projections before injection, and contact of the needle tip with bone and the resistance felt on injection were used as additional indicators of placement. Only the anteroposterior image was stored. No contrast medium was used. Local anesthetic was used only along the skin and subcutaneous needle track and was not mixed with the PRP or injected into the defect itself. Approximately 15 minutes after the injection, f-ESWT was applied to the same marked sites.

**Figure 4:**
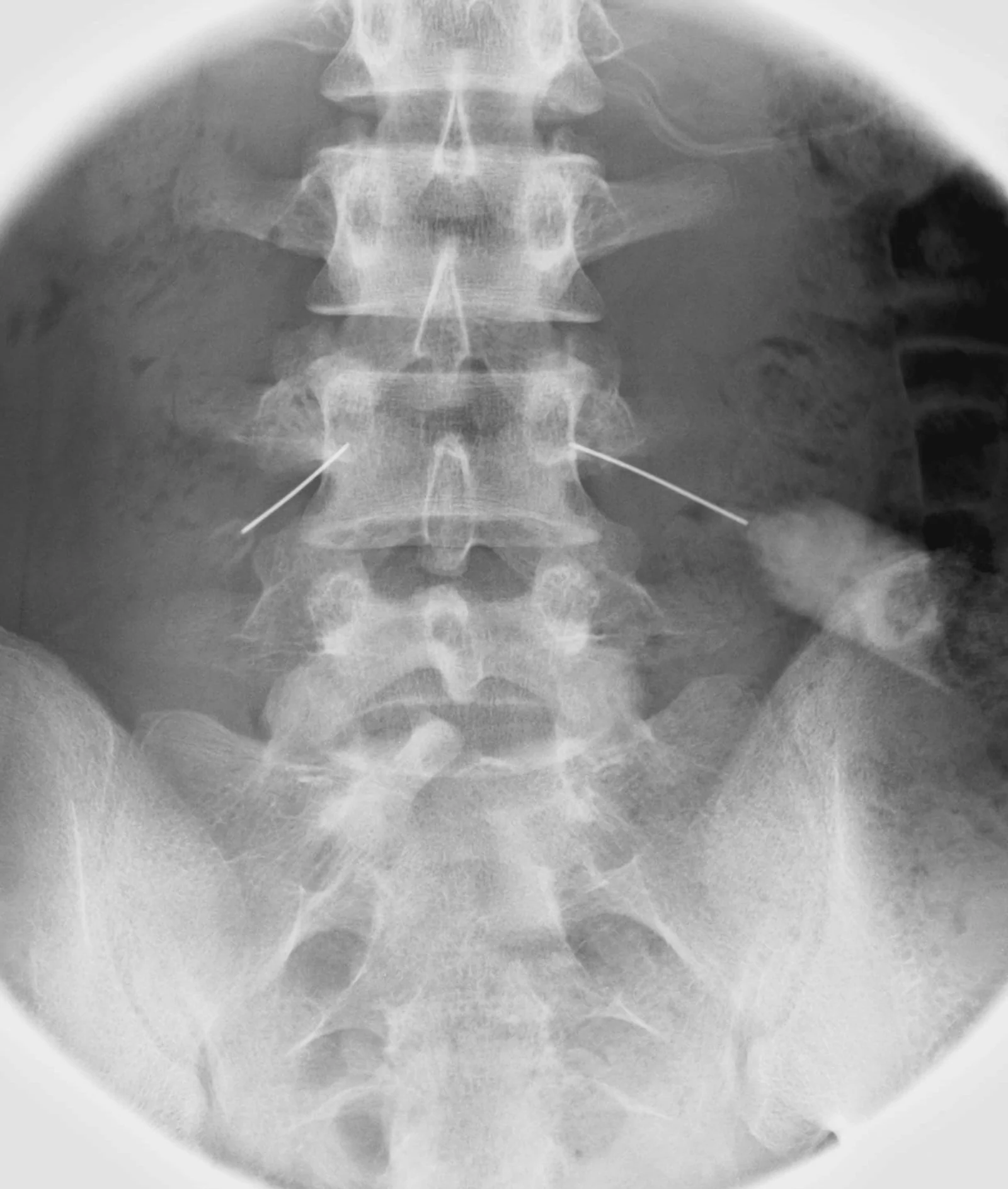
Fluoroscopic image (anteroposterior view) obtained during platelet-rich plasma injection Two needles are in place, the tip of each positioned at the L4 pars interarticularis defect on the corresponding side. A total of 6 mL of platelet-rich plasma was injected, approximately 3 mL per side. Needle position was confirmed in both the anteroposterior and the lateral projections; only the anteroposterior image was stored.

Immediately after the procedure, the patient reported injection-site pain rated 6 of 10 on an 11-point numerical rating scale [14]; this resolved without specific treatment. No other adverse events occurred. There were no neurological deficits, radicular symptoms, or signs of infection at any point during follow-up. No safety monitoring beyond the confirmation of the absence of radiating leg pain during each application and clinical follow-up was specified in advance.

The rigid orthosis was continued after treatment. CT performed approximately two months after the start of treatment showed bony bridging progressing on both sides compared with the pretreatment study. At that point, a partial return to gymnastics training was permitted, and the rigid orthosis was continued for activities of daily living. Radiographs at approximately three months after the start of treatment showed a residual fissure without diastasis on both sides; the rigid orthosis was discontinued for activities of daily living as well, and the training load was increased. The rigid orthosis had therefore been worn for approximately six months in total from the initial visit, and for three months from the start of treatment. A full return to gymnastics training was begun four months after the start of treatment. Skills involving lumbar extension and rotation were reintroduced last, and progression throughout was guided by the absence of pain rather than by a formal return-to-sport test battery. The return-to-sport milestones were recorded contemporaneously by the trainer accompanying the athlete's team.

Bony union was assessed on thin-slice CT sagittal reconstructions, with axial images used as a supplement, and was defined as continuous trabecular bridging across the pars defect; a residual lucency was permitted. This follows the operational criterion of Ohba et al., in whose sagittal classification, a lesion showing a fissure line is regarded as united when it resolves to a lucency only or to a normal appearance [1]. CT performed approximately five months after the start of treatment demonstrated continuous trabecular bridging across both pars defects, with a thin residual lucency remaining ventrally on each side; both lesions were therefore classified as united (Figures 3C, 3F, 3I). Union was also assessed retrospectively by the same three authors, each again reading the images without reference to the others' readings, and their assessments were concordant; no adjudication by an independent or blinded reader was performed. From five to six months after the start of treatment, the patient was performing complete routines on all six apparatus in training, without recurrence of low back pain. He did not return to competition during the observation period, because no event for which he was eligible was held during the off-season; complete routines on all six apparatus in training therefore represent the highest level of loading documented.

The patient trained without restriction for approximately four to five months, including complete routines on all six apparatus, without recurrence of low back pain, tenderness over the pars interarticularis, or pain on lumbar extension. Approximately nine months after the start of treatment, he sustained an acute low back injury during practice, landing from the horizontal bar with the lumbar spine forced into hyperextension. Radiographs obtained the following day showed no fracture and no sign of recurrent spondylolysis. MRI five days after the injury showed no high signal on fat-suppressed T2-weighted images at either L4 pars interarticularis (Figure 5A), and CT-like reconstructions confirmed that both pars defects remained united; the residual lucency seen at five months had largely resolved (Figure 5B, 5C). There was no neurological deficit; the pain was attributed to a soft-tissue injury, and sport was not restricted.

**Figure 5:**
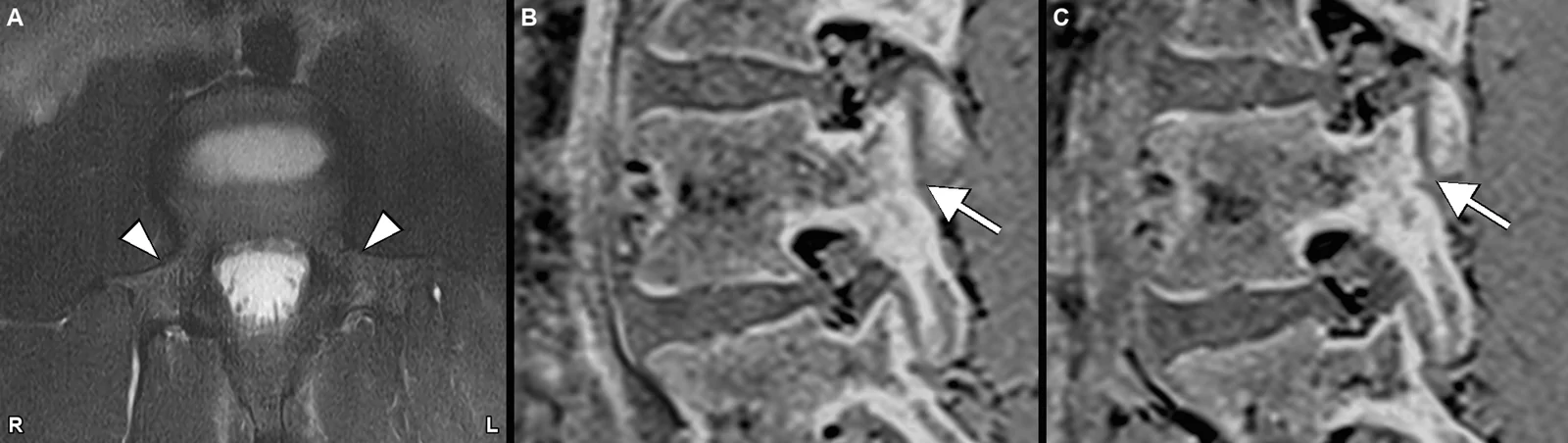
Magnetic resonance imaging nine months after the start of treatment Images obtained after an acute lumbar hyperextension injury sustained on landing, at which time the patient had been training without restriction for four to five months. (A) Axial fat-suppressed T2-weighted image at the level of the L4 pars interarticularis, showing no high signal intensity on either side. (B) Right parasagittal CT-like reconstruction. (C) Left parasagittal CT-like reconstruction. The arrowheads in the axial image (A) and the arrows in the parasagittal images (B, C) indicate the L4 pars interarticularis. R and L denote the patient's right and left. Both pars defects remained united. CT: computed tomography

The clinical course is summarized in Table 1 and Figure 6. Three outcomes are distinguished in this report: radiographic union, confirmed on CT at five months and still present on MRI with CT-like reconstructions at nine months; resolution of symptoms, with no recurrence of low back pain, pars tenderness, or pain on lumbar extension during unrestricted training; and return to sport, which was limited to unrestricted training because no competition was held during the observation period.

**Figure 6:**
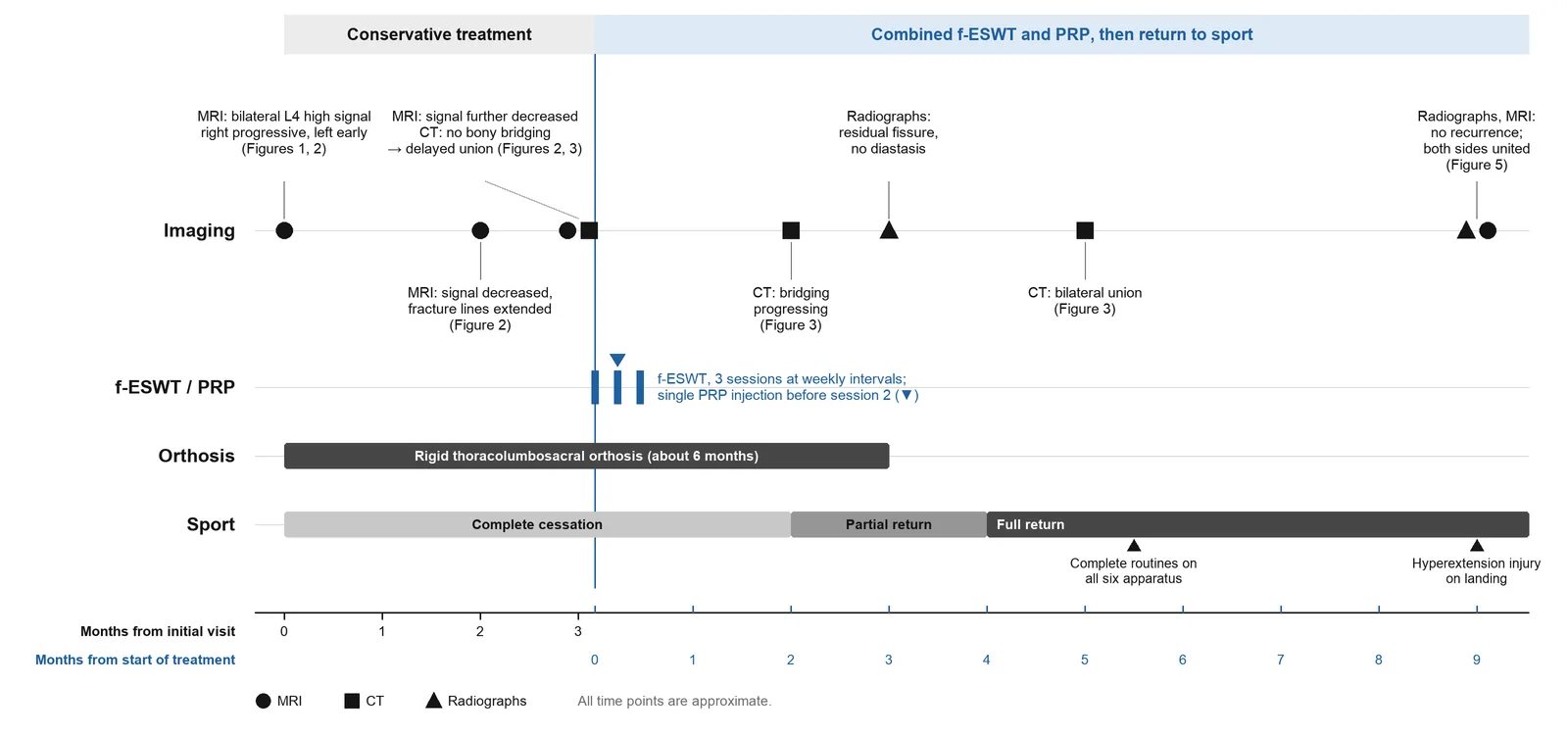
Timeline of the clinical course Time is shown in months from the initial visit (black) and from the start of combined treatment (blue); all time points are approximate. f-ESWT: focused extracorporeal shock wave therapy; PRP: platelet-rich plasma

## Discussion

In this patient, bilateral L4 spondylolysis remained ununited after approximately three months of complete cessation of sport and rigid bracing, with the right lesion at the progressive stage and the left approaching the terminal stage. Bony bridging appeared within two months of the addition of combined PRP and f-ESWT, and bilateral union was confirmed at five months, with full return to unrestricted gymnastics training. To our knowledge, and within the databases searched, this is the first report of PRP injected at a pars interarticularis defect in lumbar spondylolysis, and the first report of PRP combined with ESWT for this condition. It is to be noted that, because the classification of the left lesion as approaching the terminal stage is central to the interpretation of this case, the stage was also assessed independently by three of the authors (LI, NE, KY) for this report, to show that the classification did not rest on the judgment of a single reader, and their assessments were concordant for both sides.

The rationale for combining the two modalities lies in their complementary mechanisms. ESWT is thought to reactivate the fracture healing cascade through mechanotransduction: the shock wave produces microtrauma in existing callus, promotes the release of bioactive factors, rebalances osteoblastic and osteoclastic activity, and induces neovascularization at the fracture site [6]. PRP supplies precisely the growth factors that this cascade depends on. The sequence in which the two are applied is therefore not arbitrary. Seabaugh et al. showed, in an in vitro study of equine PRP, that applying shock waves significantly increased the release of transforming growth factor-β1 (by 33-46%) and of platelet-derived growth factor-BB (by 190-219%) compared with untreated PRP [15]. This suggests that ESWT can act as an activating stimulus for PRP already present at the treatment site, although the observation was made in vitro in equine PRP and the extent of activation in vivo is unknown. PRP was accordingly injected shortly before the second f-ESWT session so that shock wave exposure occurred while the PRP was present at the treatment site.

The energy setting was chosen on the basis of the intended biological effect on bone. A best-practice review of ESWT in musculoskeletal medicine notes that higher energy levels provide more benefit in the treatment of calcific tendinopathy and disorders of bone [16]. In practice the level was raised stepwise at the first session within the patient's pain tolerance, as described above, because an energy that cannot be tolerated cannot be delivered for the full number of shocks. The level of 0.27 mJ/mm² is the maximum energy flux density available on the device as marketed in Japan, and it was reached at the first session within the patient's tolerance. Because this exceeds the 0.20 mJ/mm² limit used in immature athletes discussed below, the decision to accept it rested on the depth of the focal zone relative to the vertebral body, as set out in the safety discussion. A leukocyte-poor preparation was used; according to the manufacturer, the ACP system yields a platelet concentration of approximately two to three times that of whole blood, although the concentration was not measured in this patient. A product in this range was chosen rather than a more highly concentrated one because in vitro data indicate that osteoblast proliferation peaks at a platelet concentration of approximately 2.5 times that of whole blood and declines at higher concentrations [17], and because a lower draw volume is preferable in an adolescent patient. The same system was used in the most closely comparable combined report [9].

The principal alternative explanation for the outcome is spontaneous union. Ohba et al. explicitly noted that many patients whose CT showed no union at three months went on to unite [1], and this must be acknowledged. However, that observation was made in a cohort of all newly diagnosed patients, in which patients whose lesions were incomplete fractures only (stages I and II, 30 of 40) predominated. Two comparisons are more relevant to this patient. First, at the initial visit, the lesions corresponded to a complete fracture on the right with an incomplete fracture on the left and acute signal change on both sides, the configuration classified as stage IIIb by Ohba et al. (a complete fracture corresponds to the progressive stage and an incomplete fracture to the early stage of the classification used above) [1]. In their series, all three stage IIIb cases with bilateral acute signal change were classified as pseudarthrosis rather than as showing union or a favorable course on the CT obtained at three months, although this subgroup is small. Whether any of those three lesions united later is not reported, so the series shows only that the three-month appearance in the present patient was typical of this configuration. Second, Iwata et al. treated newly diagnosed lesions with f-ESWT from the time of diagnosis and reported union in 76% (16 of 21) of progressive lesions but in only 10% (1 of 10) of terminal lesions, with a lower union rate when a contralateral lesion was present (92% [22 of 24] without versus 70% [28 of 40] with a contralateral lesion, p < 0.05) [2]. The present patient differs from that cohort in a direction that makes union less likely rather than more, because f-ESWT was begun only after three months of complete cessation of sport and rigid bracing had failed to produce any radiographic progression toward union.

Hatakeyama et al. reported that only one of four bilateral delayed-union cases treated with ESWT achieved union on both sides, and that in those cases every side that failed to unite was at the terminal stage [7]. The left lesion in the present patient, which was approaching the terminal stage, is therefore the lesion for which the expected probability of union was lowest. The imaging course also argues against simple continuation of a healing trajectory: over the three months of full conservative treatment before the intervention, the fracture lines extended rather than consolidated, and bridging appeared only after the intervention. This temporal relationship is suggestive but not conclusive, and in a single patient spontaneous union cannot be excluded. Moreover, rigid bracing and restriction of sport continued during and after the intervention, and PRP and f-ESWT were given concurrently. The union observed therefore cannot be attributed to either modality alone or definitively to the combination; it is reported here as an association in time, not as evidence of treatment efficacy.

The observed course is consistent with previous reports of combined PRP and ESWT for impaired bone healing. Omodani reported a 15-year-old track-and-field athlete in whom a stress fracture of the proximal phalanx of the great toe had not united after three months of conservative treatment [9]. A single session of ultrasound-guided PRP injection (leukocyte-poor, 4 mL prepared from 15 mL of whole blood; 2 mL at the fracture site and 2 mL into the metatarsophalangeal joint) was followed immediately by f-ESWT (0.25 mJ/mm², 2,500 shocks), which was repeated every two weeks; complete union was achieved at six weeks, and the athlete in their report returned to competition. In a retrospective study, Cen et al. compared PRP alone with PRP plus ESWT in 60 patients with aseptic nonunion of long bones, of whom 55 completed follow-up, and reported a union rate of 92.6% with a mean time to union of 16.3 weeks in the combined group, versus 71.4% and 21.5 weeks with PRP alone [8]. Cheng et al. reported clinical healing of a refractory middle femoral nonunion that had not responded to repeated surgery, after treatment with PRP and shock wave therapy together with structured rehabilitation [10]. LeFiles et al. reported a 48-year-old triathlete with a recurrent sacral stress fracture that had not responded to conservative treatment, in whom ESWT was followed by PRP injection; the athlete returned to competitive training at eight weeks, although bone marrow edema was still present on imaging at three months [18]. The order used in their report is the reverse of the order used in the present report, which was chosen so that the shock wave would be delivered while PRP was present at the treatment site. The age of the present patient and the order in which PRP and f-ESWT were applied parallel the report of Omodani [9], although the anatomical site and the treatment interval differ. None of these reports concerned lumbar spondylolysis, and evidence for the combination at the pars interarticularis is limited to the present case.

The safety of shock wave therapy directed at the spine deserves comment. Brain or nerve tissue within the treatment focus is listed as a relative contraindication for high-energy ESWT, and high-energy treatment should not be directed at neural tissue, including the spine [16]. An epiphyseal plate within the treatment focus is likewise listed as a relative contraindication. High-energy shock waves applied to immature bone have been reported to accelerate growth of the epiphyseal cartilage and to increase the length of long bones, and for that reason Omodani limited the energy flux density to 0.20 mJ/mm² or less in a series of 90 gymnasts aged 10-25 years (114 sites) treated for epiphyseal disorders, stress fractures, and other injuries; this level is regarded as low-energy, and limb length has been reported to remain unchanged in the long term after low-energy treatment [19]. The energy used in the present case, 0.27 mJ/mm², exceeds that limit. It is also above the 0.15-0.25 mJ/mm² used by Iwata et al. in 46 patients aged 8-24 years [2], although below the maximum of 0.50 mJ/mm² used by Hatakeyama et al. in delayed-union cases [7]. It lies at the upper end of the medium-energy range, which the best-practice review cited above defines as 0.08-0.28 mJ/mm², with the high-energy range beginning above 0.29 mJ/mm² [16].

The concern underlying the epiphyseal-plate contraindication is the effect of shock waves on growth cartilage. The target in the present patient was the pars interarticularis, a posterior element. The coupling pad placed the focal point at a depth of 30 mm, with a focal zone extending from 15 to 50 mm and a focus size of 5 × 5 × 35 mm; the pars interarticularis lay approximately 45 mm from the skin surface on CT, within that focal zone. The posterior margin of the vertebral body, by contrast, lay approximately 60 mm from the skin surface, beyond the 50-mm limit of the focal zone; neither the vertebral body nor its ring apophysis therefore lay within the focal zone. Retrospective assessment of the pretreatment CT showed that both the superior and inferior ring apophyses of L4 were at stage 3 (incomplete fusion) according to the classification of Costa et al. [20], indicating that the lumbar spine was not yet skeletally mature at the time of treatment. The position of the focal zone relative to the vertebral body is therefore directly relevant to this patient. As noted above, the shock wave was never aimed into the spinal canal, and the absence of radiating leg pain was confirmed throughout each application; these are the same precautions described by Iwata et al. [2].

The 5,000 shocks per session were divided between the two sides, so that each side received 2,500. Iwata et al. [2] and Hatakeyama et al. [7] delivered 3,000 shocks per session; neither report states whether a bilateral case received 3,000 shocks per side or per session, so the two protocols cannot be compared on a per-side basis. Sessions were held at approximately weekly intervals, whereas both of those groups treated every two weeks, so that the number of shocks delivered per unit time was higher in the present case. A weekly interval is also the protocol used in a reported series of radial shock wave therapy for nonunion of superficial bones, in which shocks were delivered once weekly for a mean of three sessions [21]; to our knowledge, no study has compared weekly with two-week intervals for bone healing. A network meta-analysis of randomized controlled trials of adjuvant treatments for fracture nonunion and delayed union reported a higher risk of adverse events with ESWT than with control (odds ratio 4.90, 95% confidence interval 1.38-17.43) [22]. In the present case the only adverse event was transient pain at the PRP injection site, and no neurological complications occurred. Ossification of the ligamentum flavum, a theoretical concern raised by Iwata et al. for shock waves directed toward the spinal canal [2], was not seen on the imaging obtained during follow-up, although no examination specific to that question was performed and the observation period was nine months. It should be noted that energy flux density values are not strictly comparable between devices, because the definition of the focal zone and the focal volume differ, so numerical comparison with previous reports using other devices is only approximate.

Limitations

This report has several important limitations.

First, it describes a single patient in whom two interventions were applied concurrently, so the individual contributions of PRP and f-ESWT cannot be determined. Second, the rigid orthosis was worn throughout the treatment period and until three months after the start of treatment, and sport remained completely restricted until two months after the start of treatment, so the intervention was added to, and not substituted for, ongoing conservative treatment. Third, spontaneous union cannot be excluded, as discussed above. Fourth, the platelet concentration of the PRP preparation was not measured, and vitamin D status and bone turnover markers were not assessed. Because the biological composition of PRP may influence its effect, the unmeasured platelet concentration limits the reproducibility of the treatment. Fifth, the last CT was obtained at five months, approximately one month after the full return to gymnastics training had begun and at approximately the same time as the athlete began performing complete routines on all six apparatus, so that no CT obtained after a longer period of unrestricted loading is available. Union was still present at nine months, after four to five months of unrestricted training and after an acute hyperextension injury, but that confirmation rests on a single MRI with CT-like reconstructions rather than on CT, and no follow-up beyond nine months is available. Nine months is also too short to evaluate the durability of union or any long-term effect of shock wave exposure on the immature spine.

Sixth, pain, lumbar range of motion, and function were not recorded with quantitative or validated measures at baseline or during follow-up, and return to sport was guided by the absence of pain rather than by a formal return-to-sport test battery. Return to competition could not be assessed, because no eligible event was held during the observation period, and unrestricted training should not be equated with competitive performance. Conclusions about functional recovery are limited accordingly. Seventh, although needle position was confirmed fluoroscopically in two projections, no contrast study was performed, and the volume injected on each side exceeded the capacity of the defect; the PRP is therefore likely to have been distributed both within and around the pars defect. Finally, skeletal maturity was not assessed before treatment.

A comparative design is needed before any conclusion about added benefit can be drawn. A practical approach would be to begin with f-ESWT alone and to add PRP only in patients whose union does not progress after a defined interval, which would allow monotherapy and combination therapy to be compared within the same cohort.

## Conclusions

In a 16-year-old competitive gymnast, bilateral L4 spondylolysis remained ununited after approximately three months of complete cessation of sport and rigid bracing, with the right lesion at the progressive stage and the left lesion approaching the terminal stage. The addition of a single PRP injection at the bilateral pars defects combined with three sessions of f-ESWT was followed, without neurological complications, by bilateral bony union at five months and, by five to six months, by a return to complete routines on all six apparatus in training without recurrence of low back pain. The only adverse event was transient injection-site pain.

Because both modalities were applied concurrently in a single patient and conservative treatment was continued in parallel, the contributions of PRP and f-ESWT cannot be separated and spontaneous union cannot be excluded. Nevertheless, given the low rate of union reported for bilateral delayed-union lesions treated with ESWT and, in particular, for lesions at or approaching the terminal stage, and given that the fracture lines had extended rather than consolidated during the preceding three months of conservative treatment, combined PRP and f-ESWT may have promoted union in this patient. This single case therefore provides a hypothesis-generating observation, not evidence of treatment efficacy; further cases and comparison with f-ESWT alone are required.
